# Machine learning for DCO-OFDM based LiFi

**DOI:** 10.1371/journal.pone.0259955

**Published:** 2021-11-23

**Authors:** Krishna Saha Purnita, M. Rubaiyat Hossain Mondal

**Affiliations:** Institute of Information and Communication Technology, Bangladesh University of Engineering and Technology, Dhaka, Bangladesh; Nanchang University, CHINA

## Abstract

Light fidelity (LiFi) uses different forms of orthogonal frequency division multiplexing (OFDM), including DC biased optical OFDM (DCO-OFDM). In DCO-OFDM, the use of a large DC bias causes optical power inefficiency, while a small bias leads to higher clipping noise. Hence, finding an appropriate DC bias level for DCO-OFDM is important. This paper applies machine learning (ML) algorithms to find optimum DC-bias value for DCO-OFDM based LiFi systems. For this, a dataset is generated for DCO-OFDM using MATLAB tool. Next, ML algorithms are applied using Python programming language. ML is used to find the important attributes of DCO-OFDM that influence the optimum DC bias. It is shown here that the optimum DC bias is a function of several factors including, the minimum, the standard deviation, and the maximum value of the bipolar OFDM signal, and the constellation size. Next, linear and polynomial regression algorithms are successfully applied to predict the optimum DC bias value. Results show that polynomial regression of order 2 can predict the optimum DC bias value with a coefficient of determination of 96.77% which confirms the effectiveness of the prediction.

## Introduction

In the modern world, the demand for high-speed wireless data is increasing at a rapid speed. Currently, wireless fidelity (WiFi) is used to provided high data rate wireless Internet. WiFi utilizes the frequency of the radio waves to transmit data and its data transmission rate ranges up to several hundreds of megabits per second. However, the radio frequency (RF) spectrum is becoming more congested, leading to an increase in the cost of spectrum licensing. Moreover, radio waves have some limitations including lack of security and the problem of interference. To address these issues, light fidelity (LiFi) has been studied in recent days. LiFi is a form of visible light communication technology that uses visible light for data transmission at the downlink. It can use infrared light for uplink communication. LiFi enables data communication along with room illumination. It is a wireless optical network technology and is considered as supplementary to WiFi. In LiFi, data are modulated onto the light intensity of optical modulators at the transmitter side. Light-emitting diodes (LEDs) and laser diodes are considered as optical modulators. On the other hand, the received data is demodulated by direct detection at the receiver. Photodiodes are considered for transforming the light signal into an electrical signal. It is a full wireless networked scheme with a handover option among optical attocells. A LiFi scheme allows user mobility and enables bidirectional communication. This can provide point to multipoint data transmission. The advantage of LiFi lies in the use of large uregulated bandwidth in the optical spectrum. Since the frequency spectrum of light waves is much higher than the RF band, LiFi has data rates up to hundreds of gigabits per second. In LiFi, the data-carrying signal does not pass the opaque walls, allowing confinement of the signal within a room. This contributes to managing interference and ensuring physical security. Furthermore, LiFi technique does not have electromagnetic interference with electrical equipment. Hence it is suitable for sensitive scenarios, including hospitals and airplanes.

The sixth-generation (6G) wireless communication is supposed to include communication in the terahertz band and support heterogeneous networks [1, 2]. Moreover, 6G will provide higher spectral efficiency, privacy, energy efficiency, customization and intelligence compared to 5G. Furthermore, 6G is expected to provide self-aggregation and self-configuration, and to enable intelligent radio in addition to cognitive radio. LiFi can be a potential candidate for 6G as it promises for secured high data rate communication in the license free terahertz band [1, 2].

The modulation techniques used in LiFi can be classified as single carrier and multi-carrier systems. In a single carrier system, on/off keying (OOK), pulse position modulation (PPM), variable pulse position method (VPPM) are used. The disadvantage of using single carrier system is the lower spectral efficiency [3]. If the spectral efficiency is low, the data rate will also be low. In multi-carrier systems, there are several modulation schemes such as asymmetrically clipped optical OFDM (ACO-OFDM), DC biased optical OFDM (DCO-OFDM), asymmetrically clipped DC biased optical OFDM (ADO-OFDM), hybrid diversity combined OFDM (HDC-OFDM), and hybrid noise cancelled OFDM (HNC-OFDM). Since DCO-OFDM uses a DC bias, it has less optical power efficiency than ACO-OFDM [4–6] for a number of scenarios; for instance, for constellations ranging from 4-QAM to 256-QAM [6]. However, for large constellation sizes for example 1024-QAM and 4096-QAM, DCO-OFDM is more efficient than ACO-OFDM in terms of optical power [6, 7]. In DCO-OFDM, a DC bias is applied to the bipolar OFDM signal to make the signal positive and the remaining negative values are clipped causing clipping noise. However, if a large DC bias is applied to reduce the clipping noise, the average optical power increases. Hence, the DC bias in DCO-OFDM must be chosen carefully.

The existing state of the art methods of choosing DC-bias are mostly optimization-based methods. In [8–11], the optimum DC biased value has been calculated by using optimization method for minimizing mean squared error (MMSE), power consumption, clipping distortion, etc. However, finding an optimized DC bias value using a machine learning (ML) algorithm is still unexplored. The advantages of ML algorithms over optimization methods are that ML algorithms can be more computationally efficient. Although ML is being considered in traditional RF wireless communication aspects, there is little work of ML in the context of OWC or LiFi. For the case of finding DC bias of DCO-OFDM based LiFi, ML can be used to identify patterns, distribution and trends in the data samples of a DCO-OFDM system. In contrast to traditional mathematics, ML can have a number of advantages in finding the optimum DC bias. ML uses intelligence to identify patterns in the data having a feature of quick self-improvement with the inclusion of new samples. In other words, ML enables recalibration based on data trend or adaptive learning. Unlike deep learning algorithms applied to image datasets, ML algorithms applied to numeric datasets usually do not require high training time, and high computational hardware resources. Even now-a-days ML is used in internet of things (IoTs) and edge devices which have low power and resource constraint. Moreover, the computation power of computing devices is improving continuously which is making ML algorithms run faster. For the particular case of ML for finding DC bias of DCO-OFDM, a numeric dataset is used and hence the computational complexity is not a major concern.

In this paper, we have used ML algorithms to find optimum DC bias value for DCO-OFDM. We identify the important features from the DCO-OFDM signal and train the model using several ML algorithms to find the optimum value of DC bias resulting in a low bit error rate (BER). The major contributions of this work are the following.

- This paper, for the first time, applies ML algorithms to find the DC bias value for an optical OFDM based LiFi. The most important factors that contribute to optimum DC bias value are also identified using ML.
- The effectiveness of different ML regression algorithms in finding the DC bias of DCO-OFDM is evaluated.

The rest of the paper is organized as follows. Next, a discussion is provided on the the existing work in the field of DCO-OFDM based LiFi. The next section analyzes how to find optimum DC-bias using ML algorithms. Results and discussions are then provided. Finally, concluding remarks are reported.

## Background and related work

### Description of DCO-OFDM based LiFi

DCO-OFDM can only transmit non-negative signals and it can carry both odd and even sub-carriers. In DCO-OFDM, a DC bias is applied to the bipolar signal and negative peaks are clipped at zero. This clipping creates clipping noise which can affect all the sub-carriers of DCO-OFDM.

**Fig 1:**
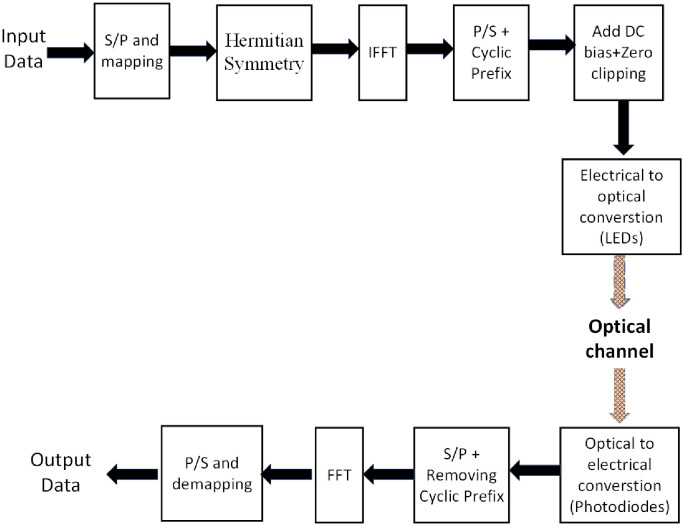
Block diagram of a DCO-OFDM based LiFi system.

Fig 1 shows that DCO-OFDM transmitter contains two extra modules compared to the conventional OFDM transmitter. One is Hermitian symmetry which is used to obtain only real-valued time-domain signal. The other one is the addition of a DC bias followed by the clipping of any negative components. At first, input data are converted from serial to parallel (S/P). The data are then mapped to complex data symbols as input to the inverse fast Fourier transform (IFFT). The complex data are denoted by *X* = [*X*_0_, *X*_1_, *X*_2_, …, *X*_*N*−1_]. The term *X* is constrained to have Hermitian symmetry and the two elements *X*_0_ and *X*_*N*/2_ are set to zero as *X*_0_ = *X*_*N*/2_ = 0 [6]. This can be expressed as follows.

$$\begin{array}{c} X_{k}=X_{N-k}^{*}\;for\;0<k<N/2 \end{array}$$ (1)

The Hermitian symmetry assures that the output of IFFT is real. The signal is then transformed from parallel to serial (P/S). The resultant real-valued DCO-OFDM signal is given by

$$\begin{array}{c} x_{n}=1/N\sum_{k=0}^{N-1}X_{k}\;e^{j2\pi kn/N}, \end{array}$$ (2)

where *X*_*k*_ is the information symbol on the *k*_*th*_ subcarrier. Then the real OFDM signal should add a DC bias. As the DCO-OFDM signal is bipolar, it has negative peaks. So, to eradicate the negative peaks, a DC bias level *K*_*DC*_ is added to the time domain signal, where the term *K*_*DC*_ is given by

$$\begin{array}{c} K_{DC}=\mu \sqrt{E\{x{\left(t\right)}^{2}\}} \end{array}$$ (3)

where *μ* is the proportional constant and *K*_*DC*_ is represented as a DC-bias of 10*log*_10_(*μ*^2^ + 1) dB.

Next, a cyclic prefix is added for protecting the OFDM signal from inter-symbol interference. An OFDM signal has a very high peak-to-average power ratio, this means the peak value is much greater than the average value. Hence, some of the positive and negative peaks are very high in OFDM. A high DC bias is required to remove all the negative peaks. However, the high DC bias is very expensive in terms of optical power. Hence, an optimum DC bias is required for DCO-OFDM.

Next, the optical signal is transmitted over wireless optical media. The optical signal experiences the channel impulse response and noise. Similar to the work in the literature [6], the link is considered to be a directed line-of-sight one affected by only signal-independent noise. This noise is usually caused by background light and receiver preamplifier. It can be modeled as additive white gaussian noise (AWGN). It is added to the incoming signal in the electrical domain after the signal is transformed to electrical by the photodetecting elements. In order to recover the transmitted data, the receiver does operations that are inverse to the actions of the transmitter block.

### Related work

Artificial intelligence (AI) can be defined as human-like intelligence demonstrated by machines or computers. ML is a subset of AI and refers to machines learning from data with explicit programming. In other words, ML involves algorithms that improve via new user data and with experience. In ML, a model is developed with the use of sample or training data, and this model is later used to predict decisions for future unknown data samples. The model improves with the inclusion of new data samples without a change in programming. Classification and regression are two major types of ML algorithms. In the case of regression, a dependent variable is evaluated based on an independent variable. On the other hand, in classification, the dependent variable has discrete values, for example, binary, tertiary, etc. Currently ML is applied to many scenarios including data security, personal security, health care, financial trading, transportation, and analysing complex and big data in industries. Next-generation wireless communication needs to support high data transfer rates to a large number of connected devices of various types. The use of ML can accelerate to achieve this goal. Recent studies show that ML can be used in several cases, for instance, base station (BS) association, power control and switching control, and management of spectrum, backhaul, network, cache and mobility [12–14]. Moreover, ML is being studied for the case of telecommunication routing and localisation [13, 14], and for wireless channel modelling and estimation [12, 15]. In [16], a naïve Bayes classifier is used to reduce spectral sensing time.

There are several papers on ML algorithms and DCO-OFDM. In [17], the authors proposed to reprocess fractional frequency in optical attocells in a special visible light communication scheme. In [18], Cheng Chen et. al. analyzed downlink transmission in optical attocell network based on DCO-OFDM for a system level framework. Here, DCO-OFDM had been used in multi-user access scheme to obtain high spectral efficiency in the downlink transmission where DC bias had been mobilized for illumination purposes. The authors in [9] presented an optical scaling scheme to maximize signal to noise ratio (SNR) where DC bias value would be fixed for a wanted dimming value. One study [19] aimed to employ profitable clipping method in DCO-OFDM for reducing BER by using Bussgang theorem and Gaussian distribution. However, this paper did not discuss DC bias. In [20], adaptive DCO-OFDM was considered for underwater visible light communication to find out maximum throughput and low BER through subcarrier allocation and the selection of modulation size. In [21], a discussion was provided about HNC-OFDM which was composed of ACO-OFDM and DCO-OFDM. Furthermore, HNC-OFDM was compared with other forms of OFDM. Although ML had not been used for selecting the DC bias for DCO-OFDM based LiFi, it had been widely studied in other contexts. The authors in [22] reported a clinical decision support system for helping patients with low back pain. For this research, they applied supervised ML methods with three classification models namely decision tree, random forest and boasted tree. In [23], the authors studied the impact of process-level information using several ML algorithms including linear regression, logistic regression, support vector machine (SVM) and artificial neural networks. In [24], a dataset of 2015 medicare samples was classified and it was shown that supervised algorithms are suitable for fraud detection for medicare systems. In [25], ML was used to analyze various clinical parameters, data analysis, intelligent alarming results using ML algorithms. In [26], the effectiveness of different ML methods was discussed. In [8, 10], the optimum DC biased value was evaluated using numerical method formulated on minimizing mean squared error (MMSE) through optimization method. In [10], optimum DC-biased value was analyzed using power optimization method by minimizing power. In [11], the authors figured out optimum DC biased value by using MMSE equalizer. In [9], the authors explored optimum DC biased value by an optimization method by minimizing mean squared value for cutting down the consequence of clipping distortion. In the above-mentioned studies, the authors mainly focused on optimization methods based on power and minimum mean square root for finding the optimum DC biased value. However, none of these studies used a ML algorithm for finding the optimum DC bias value. The advantage of ML algorithm over optimization algorithms is less computation time. Moreover, the application of ML helps in finding the optimum DC bias of DCO-OFDM. When DCO-OFDM system, uses an optimum DC bias, then the system becomes more power efficient compared to a system using too high or too low DC bias. In other words, for a given power level, a DCO-OFDM system with optimum DC bias has a lower BER than a system with a non-optimal bias [8]. Hence, this paper focuses on the application of ML in finding the required DC bias for DCO-OFDM.

## ML to find optimum DC-bias

### Data generation

To use ML algorithms for finding the optimum DC bias, a dataset was generated for DCO-OFDM systems. For data generation, MATLAB tool was used. The DCO-OFDM signal generation depends on a number of parameters including the size of constellation points (M) that denotes k number of bits per symbol, energy per bit to noise spectral density (*E*_*b*_/*N*_0_). Firstly, the data was mapped to the quadrature amplitude modulation (QAM) constellation points. Hermitian symmetry was maintained for the complex constellation points. Then, an IFFT operation was performed on the constellation points. Because of the Hermitian symmetry, the output of the IFFT was real valued signal. The output of the IFFT was a bipolar OFDM signal. A DC bias was then added to the bipolar OFDM signal, and any remaining negative components were clipped to form a unipolar DCO-OFDM signal. Multiple OFDM signals were generated for different parameters. Then, from these OFDM signals, the mean, the min (minimum), the max (maximum), the standard deviation (std) and the BER values were calculated. With these values, the dataset was generated.

**Algorithm 1**: Algorithm of Data Generation

**Require**: *M*: Signal constellation size, *b*: Bias values, *N*: Number of subcarriers, *N*_*bits*_: Number of bits to be processed

**Ensure**: *min*: Minimum value of the OFDM signal, *max*: Maximum value of the OFDM signal, *std*: standard deviation of OFDM signal, *mean*: mean value of OFDM signal, *BER*: Bit error rate

1: *k* ← log_2_ *M*

2: *SNR* ← *EbNo* + 10 * log_10_ *k*

3: *snrLen* ← Calculating the length of (SNR)

4: *hMod* ← Generate *QAM*(*M*)

5: **for** *j* ← 1 to *snrLen* **do**

6:   *Totalbits* ← 0

7:   **while** *Totalbits* ≤ *N*_*bits*_ **do**

8:    *data* ← Generating random data

9:    *Mod_data* ← *modulate*(*hMod*, *data*)

10:    *Hermitian_data* ← Ensuring Hermitian Symmetry of *Mod_data*

11:    *OFDM* ← IFFT(Hermitian_data)

12:    *DCO* − *OFDM* ← (*OFDM* + *bias* + *clipping*)

13:    *Totalbits* ← *Totalbits* + (*N*/2) × *k*

14:   *mean*(*j*) ← *mean*(*DCO* − *OFDM*)

15:   *min*(*j*) ← *minimum*(*DCO* − *OFDM*)

16:   *max*(*j*) ← *maximum*(*DCO* − *OFDM*)

17:   *std*(*j*) ← *Standard Deviation*(*DCO* − *OFDM*)

18:   *BER*(*j*) ← Calculating bit errors

19: **return** *mean*, *min*, *max*, *std*, *BER*

### Using ML algorithms

By simulating the DCO-OFDM system in MATLAB, the mean, the min, the max, the std, the BER values, the constellation size M and the subcarrier number N were collected to generate the dataset. The dataset was trained using several ML algorithms to find out maximum accuracy from which the most suitable algorithm was found.

#### Linear regression

While applying linear regression methods in ML, we imported Numpy, Panda, and, Seaborn libraries. By applying Sklearn method, we imported datasets and linear model metrics for running linear regression which follows a relationship between a dependent and an independent variable. To run linear regression model in Python, we imported libraries and datasets, encoded the absolute dataset and ignored the variable data. The dataset was then divided into training and testing data. For feature selection, we used sklearn.feature_selection to import SelectKBest, f_regression for best feature selection. For the case of k = 6, we got the best six features. Linear Regression is the ML algorithm that is bottomed on the supervised model. The task was implemented by linear regression by the anticipation of a dependent value ‘*y*’ which is based on the value of dependent value ‘*x*’. The simple equation of linear regression is given below.

$$\begin{array}{c} y=ax+b \end{array}$$ (4)

For multiple variables, the equation of the linear regression is given below.

$$\begin{array}{c} y=b+a_{0}x_{0}+a_{1}x_{1}+a_{2}x_{2}+a_{3}x_{3}+\ldots \ldots ..+a_{n}x_{n} \end{array}$$ (5)

#### Polynomial regression

Polynomial regression is the configuration of regression analysis which forms a relationship between the independent value x and dependent value y of nth degrees. It is the expression of non-linear data model. It is determined to be an extraordinary case of multiple linear regression methods. The equation of the polynomial regression of *m* degree is given below.

$$\begin{array}{c} \begin{array}{c} y=k+\left(a_{00}x_{0}+a_{01}x_{1}+\ldots .+a_{0n}x_{n}\right)+\left(a_{10}x_{0}^{2}+a_{11}x_{1}^{2}+\ldots .+a_{1n}x_{n}^{2}\right)+\ldots ..\\ +(a_{m0}x_{0}^{m}+a_{m1}x_{1}^{m}+\ldots .+a_{mn}x_{n}^{m}) \end{array} \end{array}$$ (6)

**Algorithm 2: Applying ML Algorithm**

  **Input**: A training and testing dataset

  **Output**: Optimum DC bias value

1: Load the dataset using a data loading function.;

2: Divide the dataset into input and output;

3: Assign the input data to x;

4: Assign the output (DC bias value) to y;

5: Use the split method to divide the x and y into training and test data;

6: Select features from training data and get feature score;

7: Train the ML model using the selected features of training data;

8: Predict the DC bias value using the training model and test data;

## Results and discussion

### Experimentation

Our experiment had two parts. In the first part, DCO-OFDM system was simulated using MATLAB for generating a dataset. By changing the DC-biased value, the constellation size and the number of sub-carriers, a dataset was generated by taking 250 samples and 8 attributes. The records in the dataset were randomly placed. In the second part, the dataset was used in Python for the application of machine learning. The holdout method was used using the train_test_split function to divide the dataset into training and testing samples. The holdout technique randomly spits the dataset in each run. This results in different samples for training and testing phase. In the experiment, different training and testing samples were considered. In this paper, the results are shown for the case where the shuffling of the dataset was turned off which ensures fixed results for each run. With this consideration, a split value of 0.3 resulted in the first 70% of the dataset to be used as training and the remaining 30% as testing data. For implementing in Python, Jupyter notebook was used in a MacBook Air laptop which has 2.2 GHz Dual-core Intel Core i7 processor and 8 GB 1600 MHz RAM. In Table 1, the names of attributes of the dataset are mentioned.

**Table 1:** Dataset attributes.

Serial	1	2	3	4	5	6	7	8
Attribute Name	M	Mean	Min	Max	std	BER	Bias	N

### Linear regression

For the case of linear regression, the dataset was divided into 70% trained and 30% tested data samples unless explicitly mentioned. Using this data, we selected features using uni-variant feature selection method. For this method, we imported SelectKBest function. In this SelectKBest, we used F2 regression method for selecting the important features. From Table 2, the best feature is the maximum value of OFDM signal denoted as the Max. The best 5 features are the max, the std, the min, M, and the subcarrier number N. Table 3 indicates that for the case of 5 to 7 features we get the best training and testing accuracy values. Table 4 shows the coefficient values of linear regression. According to the linear equation in (5), the number of coefficients varies with the number of features. By using the coefficient values, we verified the accuracy of the model for some of the test values.

**Table 2:** Feature score of attributes.

Attribute	Feature Score
M	241.63
Mean	1.14
Min	300.39
Max	461.4
std	427.57
BER	6.11
N	101.18

**Table 3:** Training and testing accuracy with features for linear regression.

Number of Features	Training Accuracy	Testing Accuracy
1	0.66113	0.61463
2	0.66131	0.61213
3	0.66134	0.61143
4	0.66375	0.61587
5	0.82080	0.84126
6	0.82960	0.84065
7	0.82982	0.84052

**Table 4:** Coefficient of linear regression based on feature selection.

# of Feature,K	*a*_0_	*a*_1_	*a*_2_	*a*_3_	*a*_4_	*a*_5_	*a*_6_	*b*
7	-0.00178	-1.044	0.0546	0.499	1.9916	-76.8	0.0019	0.26712
6	-0.00178	0.0546	0.5	1.9931	-76.5	0.0019	N/A	0.26266
5	-0.00182	0.0806	0.517	1.9763	0.00188	N/A	N/A	0.13783
4	-3.5E-04	0.0113	0.863	-0.199	N/A	N/A	N/A	0.99383
3	0.012115	0.7653	-0.16767	N/A	N/A	N/A	N/A	1.02713
2	0.760575	-0.1870	N/A	N/A	N/A	N/A	N/A	1.02665
1	0.701422	N/A	N/A	N/A	N/A	N/A	N/A	1.02369

We measured the accuracy of the linear regression model by taking the root mean square error (RMSE) and the coefficient of determination known as R2 score. We observed RMSE and R2 scores by taking different numbers of features as shown in Table 5. Table 5 indicates that the best performance is achieved when 5 features are used, while the performance degrades when less than 5 features are considered. By taking the best five features, the highest R2-score of 0.8412 and the lowest RMSE value of 0.4271 were obtained. On the other hand, with 4 features, a low R2-score of 0.6158 and a high RMSE of 0.6644 were obtained.

**Table 5:** RMSE and R2-score in linear regression.

# of Features	RMSE	R2-square
1	0.6654	0.6146
2	0.6676	0.6121
3	0.6682	0.6114
4	0.6644	0.6158
5	0.4271	0.8412
6	0.4279	0.8406
7	0.4281	0.8405

Table 6 shows the different ratios of training and testing data samples for the best five features. The size of the testing data samples was varied from 0.1 to 0.5 of the total dataset. For each of the cases, we got R2 scores greater than 0.80. For example, at a test size of 0.3, an R2 score of 0.8412 and an RMSE of 0.4271 were obtained for the best 5 features.

**Table 6:** Results of linear regression in terms of RMSE and R2-score.

Train-Test ratio	RMSE	R2 Score	Training Accuracy	Testing Accuracy
0.1	0.4399	0.8042	0.8313	0.8042
0.2	0.4416	0.8316	0.8269	0.8316
0.3	0.4271	0.8412	0.8208	0.8412
0.4	0.4425	0.8390	0.8180	0.8390
0.5	0.4592	0.8333	0.8109	0.8333

**Fig 2:**
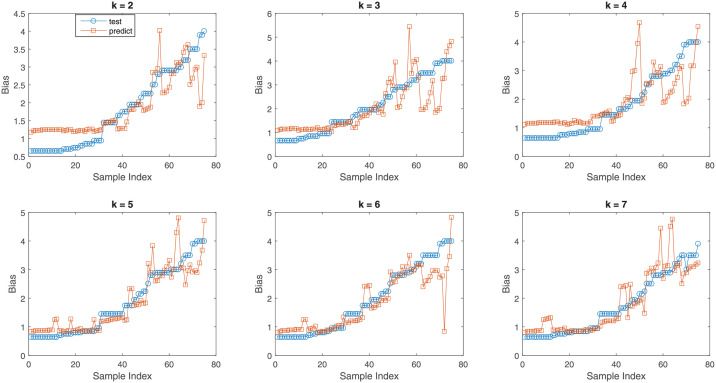
Actual and predicted bias values for linear regression.

To show the effectiveness of linear regression, we plotted the actual values of DC biased and predicted values of DC bias for different number of features. This is shown in Fig 2. The plots are for different values of features *k*. We can see from Fig 2 that for the cases considered, the tested value of DC bias and the predicted value of DC bias are aligned to a good extent. So, linear regression can be useful in predicting the appropriate DC bias value for DCO-OFDM based LiFi.

### Polynomial regression

For the case of polynomial regression, we calculated RMSE and R2 scores to find the accuracy of the model. Table 7 shows the results of polynomial regression considering 5, 6 and 7 attributes, test size varying from 0.1 to 0.3 and the degree of polynomial from 1 to 3. For the case of best 5 features, polynomial regression with degree 2 or degree 3 show comparable performance with R2 scores around 0.95. However, when 6 or 7 features are taken into consideration, polynomial regression with degree 2 has the best performance having a high R2 score for test sizes of 0.1, 0.2 and 0.3. From Table 7 it can be seen that, for the case of 6 features and for degree 2, the highest R2 score is 0.96774 with an RMSE value of 0.19253. Hence, for the generated dataset and for the cases considered, the best fit is obtained when polynomial regression of degree 2 is used.

**Table 7:** RMSE and R2-score in polynomial regression.

Test_size	Degree, n	k = 5		k = 6		k = 7	
		RMSE	R2	RMSE	R2	RMSE	R2
0.1	1	0.43991	0.80429	0.42547	0.81692	0.42510	0.81725
	2	0.22107	0.95057	0.22071	0.95073	0.22062	0.95077
	3	0.22407	0.94922	0.26790	0.92741	0.26610	0.92838
0.2	1	0.44169	0.83162	0.43523	0.83651	0.43513	0.83658
	2	0.21112	0.96153	0.19987	0.96552	0.19986	0.96552
	3	0.20861	0.96243	0.40258	0.86012	0.39459	0.86561
0.3	1	0.42710	0.84126	0.42793	0.84065	0.42810	0.84051
	2	0.20547	0.96326	0.19253	0.96774	0.19254	0.96773
	3	0.20466	0.96354	0.38034	0.87411	0.49571	0.78617

**Fig 3:**
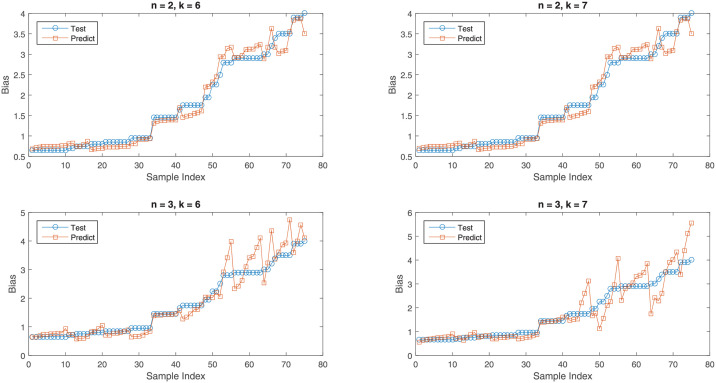
Actual and predicted bias values for polynomial regression.

Next, the effectiveness of polynomial regression is discussed by plotting the actual values of DC biased and predicted values of DC bias for different number of features. This is shown in Fig 3. The plots are for different degrees *n* and different features. We can see that for degree 2, the test value and the predicted values are best aligned as expected from the RMSE and R2 score of Table 7.

From the above discussion, it can be seen that the highest R2 scores for the case of linear and polynomial regressions are 0.8412 and 0.96774, respectively. This indicates that both linear and polynomial regression can be considered for finding the appropriate DC bias for DCO-OFDM. Moreover, polynomial regression model is more reliable in predicting DC bias value for a DCO-OFDM based LiFi. Note that the models are based on the input attributes mentioned in the dataset and the output attribute which is the DC bias value. So, for given values of input attributes: M, N, the target BER, and the mean, the min, the std, and the max values of the transmitted OFDM signal, the model can predict an appropriate DC bias value. This predicted DC bias value is optimum as it ensures an uncoded target BER close to 10^−3^. This uncoded target BER of 10^−3^ is equivalent to a BER of 10^−9^ with channel coding. Hence, with the generated dataset and the linear or polynomial regression algorithms, a practical DCO-OFDM based LiFi system can select an appropriate DC bias value that will ensure a BER lower than a target BER.

## Conclusion

This paper determines the optimal DC bias value using different ML algorithms. A DCO-OFDM based LiFi system was simulated to generate a dataset for predicting the DC bias of DCO-OFDM. Next, ML algorithms were applied to the dataset. The most important attributes of DCO-OFDM were determined that have influence on the optimum DC bias selection. Results show that linear regression and polynomial regression are reliable in predicting the optimum DC bias value when at least 5 features are considered. Results also show that for the cases considered, polynomial regression with order 2 can obtain a high R2 score of 0.96774. In the future, a larger dataset of DCO-OFDM based LiFi system can be generated. The effectiveness of the linear and the polynomial regression models have to be evaluated for the case of different datasets with large samples. Moreover, the optimum DC bias for other OFDM systems, for example ADO-OFDM should also be studied. The concept and the findings of this paper can be applied in the future to other aspects of LiFi.

## Supporting information

S1 Dataset (CSV)

S1 File (M)

S2 File (PY)

S3 File (PY)
